# Correction: DNA damage induces nuclear actin filament assembly by Formin-2 and Spire-1/2 that promotes efficient DNA repair

**DOI:** 10.7554/eLife.11935

**Published:** 2015-10-05

**Authors:** Brittany J Belin, Terri Lee, R Dyche Mullins

Belin BJ, Lee T, Mullins RD. 2015. DNA damage induces nuclear actin filament assembly by Formin-2 and Spire-1/2 that promotes efficient DNA repair. *eLife* **4**:e07735. doi: 10.7554/eLife.07735

Published 19 August 2015

In the originally published version, the title was given as: DNA damage induces nuclear actin filament assembly by Formin-2 and Spire-½ that promotes efficient DNA repair.

The title should have read: DNA damage induces nuclear actin filament assembly by Formin-2 and Spire-1/2 that promotes efficient DNA repair.

The article has now been corrected.

